# Note on the Physiological Effects of Carbonic Oxide

**Published:** 1871-04

**Authors:** A. R. Leeds


NOTE ON THE PHYSIOLOGICAL EFFECTS OF CARBONIC OXIDE.—By Prof A. R. Leeds.—I accidentally respired, some time ago, a quantity of pure carbonic oxide. The gas was contained in a quart bottle, from which I inhaled certainly less than a pint—probably a quantity not exceeding a g ll—into my lungs, previously exhausted through expiration of atmospheric air. For a moment no change of mental impressions or of bodily feelings was noticeable. The next, without any intermediate condition, I was struck senseless to the floor. Fortunately the bystanders rushed immediately forward, tore open my clothing, poured water upon my wrists and head, and applied violent friction to my limbs. The pulse had stopped beating, or beat so feebly that in the agitation of the moment it was imperceptible; the chest had ceased to expand and contract, the complexion had assumed the livid hue of death, and the temperature of the body was rapidly falling. The operation of the carbonic oxide was so immediate as to prevent the lungs from throwing off the single charge they had received, and the shock arising from the remedies employed probably enabled them to do so A slight nausea, which passed off in the course of a few hours, and a dullness and oppression in the crown of the head, lasting some time longer, were the only effects which remained after restoration to consciousness.

